# Selective Control of Attention Supports the Positivity Effect in Aging

**DOI:** 10.1371/journal.pone.0104180

**Published:** 2014-08-05

**Authors:** Laura K. Sasse, Matthias Gamer, Christian Büchel, Stefanie Brassen

**Affiliations:** Department of Systems Neuroscience, University Medical Center Hamburg-Eppendorf, Hamburg, Germany; Goldsmiths, University of London, UK, United Kingdom

## Abstract

There is emerging evidence for a positivity effect in healthy aging, which describes an age-specific increased focus on positive compared to negative information. Life-span researchers have attributed this effect to the selective allocation of cognitive resources in the service of prioritized emotional goals. We explored the basic principles of this assumption by assessing selective attention and memory for visual stimuli, differing in emotional content and self-relevance, in young and old participants. To specifically address the impact of cognitive control, voluntary attentional selection during the presentation of multiple-item displays was analyzed and linked to participants' general ability of cognitive control. Results revealed a positivity effect in older adults' selective attention and memory, which was particularly pronounced for self-relevant stimuli. Focusing on positive and ignoring negative information was most evident in older participants with a generally higher ability to exert top-down control during visual search. Our findings highlight the role of controlled selectivity in the occurrence of a positivity effect in aging. Since the effect has been related to well-being in later life, we suggest that the ability to selectively allocate top-down control might represent a resilience factor for emotional health in aging.

## Introduction

Over the past 10 years, a great amount of studies has been dedicated to explore the phenomenon of a positivity effect (PE) in older adults' information processing [1]. The PE describes an age-related increase in the ratio of positive relative to negative information processing, which can either be driven by a heightened processing of positive information, or a diminished processing of negative information [2]. For instance, older versus younger participants were found to be less distracted when responding to a dot target appearing after a negative relative to a neutral item [3]–[5] and to attend relatively more on positive [6]–[8] and less on negative items [9], [10]. Moreover, some studies demonstrated that in relation to their overall memory performance, elderly people recall a larger proportion of positive items and a lower proportion of negative items than younger adults [11], [12]. Motivated by theoretical assumptions, recent work has now begun to investigate the boundary conditions of this effect. In particular, it could be demonstrated that the occurrence of the PE depends on the degree of natural information processing permitted by the paradigm and substantially relies on cognitive resources (for reviews see [1], [2]).

The most prominent theoretical framework for the PE is provided by the Socio-emotional Selectivity Theory [13], [14]. The theory attributes the effect to a systematic shift in goal-setting behavior, occurring in the context of a limited life-time perspective. More specifically, narrowed time horizons in late life lead to a greater priority of goals about emotional meaning and well-being, which direct the allocation of cognitive resources during the processing of emotional information. In contrast, younger people, who normally perceive their lifetime as more open-ended, prioritize future-oriented goals, such as expanding knowledge and making new experiences, and consequently focus less on their emotional state. Supporting this notion, a PE can be observed also in younger adults when instructed to focus on their current emotional state [15]. In addition, the prominence of emotional aspects for younger adults appears to increase when being confronted with a limited life-time perspective [16].

It has been speculated that the shift towards emotional goals in aging is closely linked with an age-related increased engagement of emotion regulation [17]–[19]. This speculation is supported by self-reports of healthy older adults, indicating a greater access to emotion regulation strategies as well as a stronger ability to engage in goal-directed behavior [20], [21]. Moreover, experimental studies have repeatedly documented reduced distractibility by negative information [3]–[5], [22], [23] and preferred processing of positive distractors and information in older as compared to younger adults [6], [7], which was directly associated with emotional well-being [6]. More support comes from recent neuroimaging studies, demonstrating an increased engagement of ventromedial brain regions during the generation of a PE in older adults [6], [24], [25]. Ventromedial brain regions, including the anterior cingulate cortex, are assumed to be key nodes of the “emotion regulation network” [26]–[28]. In addition, medial prefrontal brain regions have been associated with self-referential processing [29], one of the discussed mechanisms facilitating a boost in attention and memory for positive items in older adults [25], [30]–[32].

There is also evidence for an age-related shift in the choice of prioritized emotion-regulation strategies for maintaining wellbeing. According to self-reports and experimental findings, older adults seem to specifically exert self-directed attention-control strategies involving selective attention and withdrawal from distressing situations when being confronted with emotional information [19], [33]. Interestingly, in contrast to younger age, such suppression strategies are not associated with enhanced psychological distress (i.e. depression, stress or anxiety) but, instead, may be an effective emotion regulation tool against the stressors experienced in later life [34].

Both selectively focusing on positive information and ignoring negative distraction probably rely on cognitive resources and the general ability of top-down control [18], [19]. In line with that, the “cognitive control hypothesis” states that elderly people selectively invest cognitive control to pursuit their emotional goals [35]. Consistent with this assumption, the PE can be diminished when attentional resources are exhausted by a secondary task [6], [10], [12], and a PE in memory is reduced in older people with lower degrees of executive control [12]. On the other hand, executive control is frequently affected by an age-related decline [36]. In fact, older adults often show worse performance than younger adults during interference tasks with neutral distractors [37]. Moreover, the “aging brain model” [38] has argued that reduced responses to negative stimuli in older adults result from a decline in arousal-sensitive brain circuits, including the amygdala (see [39] for a critical discussion). As described above, there is emerging evidence speaking against such a deficit-oriented approach. However, it is still rather unclear whether and which preserved cognitive ability might facilitate a PE in aging.

In the present study, we aimed to shed more light on the conditions and mechanisms underlying a PE in aging. To investigate the impact of voluntary selective attention on the occurrence of the effect, we analyzed young and older participants' fixation profile during a novel free-viewing eye-tracking paradigm in which images of positive, negative and neutral social scenes were pitted directly against one another in triads. As demonstrated in a recent meta-analysis, the magnitude of the PE is significantly reduced in studies that constrain elderly people's natural information processing through specific experimental instructions [1]. By presenting different emotional stimuli simultaneously, we maximized the need to engage cognitive control in order to selectively process information. To evaluate the depth of (selective) information processing, we also assessed participants' subsequent memory performance in a recognition paradigm on the following day. As described above, it has been speculated that the selective depth of information processing in older adults might be modulated by an increased tendency to process positive information in relation to oneself [31]. To follow up on this idea, we varied the attractiveness for self-referential processes in our paradigm by including images that display younger versus older adults in age-typical social scenarios (e.g. a wedding versus playing with grandchildren). Both older and younger adults have been found to be more distracted by own-age compared to other-age faces [7] and to show a preference for faces of the own age in attention [40], [41] and memory [42], all of which points to an enhanced self-referential processing [43], [44]. Finally, in order to investigate the impact of participants' general cognitive ability to exert attentional top-down control over salient distraction on the occurrence of a PE, we measured participants' performance in a visual search task. Overall, our study was designed to further illuminate the main hypotheses on the basic mechanisms underlying the PE in aging.

## Materials and Methods

### Participants

Twenty-five younger (18–30 years; *M* = 24.28; *SD* = 3.20; 10 men) and 25 older (62–78 years; *M* = 67.56; *SD* = 4.43; 12 men) adults participated in the present study. Three additional participants were excluded prior to the analyses due to difficulties in obtaining stable eye tracking data (<70% valid data in more than half of the trials). All participants had normal or corrected-to-normal vision (including color vision), and no present or previous neurological or psychiatric disorders like depression or dementia. Older participants successfully completed the neuropsychological battery of the Consortium to Establish a Registry for Alzheimer's Disease (CERAD [45]) including the Mini-Mental State Examination (MMSE, all participants >28). Participants were recruited via an online-announcement and from an existing database and were paid 10 Euro per hour for participation. The study was approved by the local ethics committee of the Medical Association, Hamburg, Germany, and all participants gave written informed consent before participating.

### Study design and tasks

There were two consecutive study days. On the first day, selective visual attention to emotional and neutral social scenes was assessed using an eye-tracking paradigm. Afterwards, participants' general ability to exert top-down control over salient distraction was measured with a visual search task. Twenty-four hours post eye-tracking, memory performance for the material was tested in a recognition paradigm. This was followed by emotional ratings of each image. Measurements were conducted in a sound-attenuated, air-conditioned and shaded room with constant illumination. The recording and programming equipment was located outside the room. Participants were tested individually while the experimenter stayed outside the room.

#### Eye-tracking paradigm

**Figure 1:**
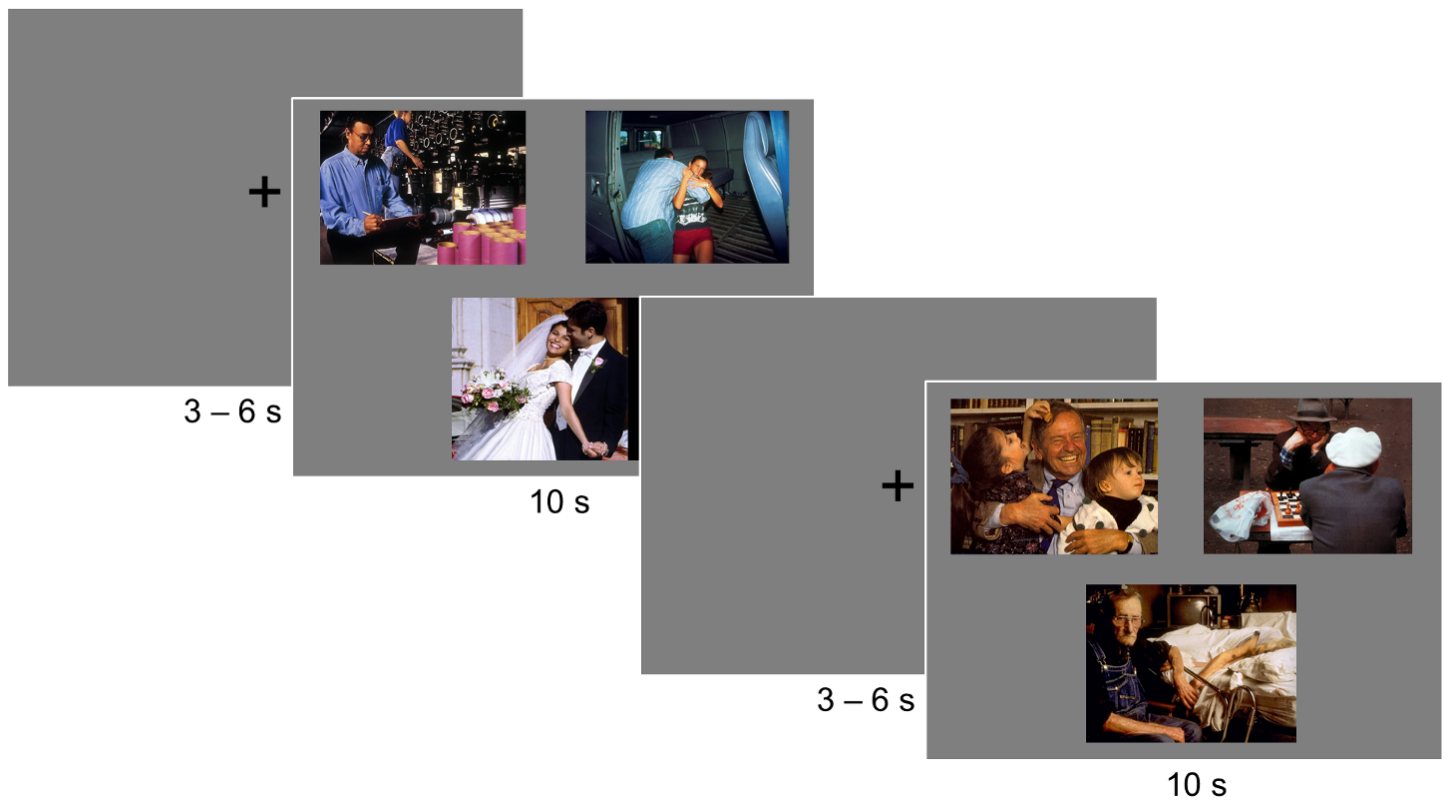
Outline of the eye-tracking paradigm. Two consecutive trials are presented, each starting with a fixation cross, followed by a display of a positive, negative and neutral social scene. The first trial includes social scenes that depict young adults in age-typical scenes, while images of the second example trial belong to the old-age category.

Stimuli of the eye-tracking paradigm consisted of 240 color photographs selected from the internet as well as from the International Affective Picture System (IAPS; [46]). All pictures had a size of 600×450 pixels. Pictures featured social situations, involving at least two persons in distinct positive, negative or neutral social interactions. Half of those pictures depicted elderly people in social situations that were thematically more relevant for their respective age (e.g. playing with grandchildren, funeral; see Figure 1) and the other half of the images showed younger people in social situations more typical for young adulthood (e.g. wedding, brawl). Affiliation of the images to the emotion and relevance categories was based on the consentaneous classification by three independent raters from our laboratory. Moreover, picture categories were confirmed by an independent sample of 20 young participants, who rated all images on a valence scale (ranging from 1 = very negative to 6 = very positive) and classified them according to their relevance for people around 30 years versus 65 years of age. In this sample, the average valence ratings were *M* = 1.73, *SD* = 0.16 for negative, *M* = 3.18, *SD* = 0.24 for neutral and *M* = 4.08, *SD* = 0.20 for positive images. The classified age-relevance of the stimuli was in accordance with the a priori grouping for 91% of the negative stimuli, for 86% of the neutral stimuli and for 87% of the positive stimuli.

Out of the 240 images, two sets of 120 stimuli were created. Participants were randomly assigned to one of the two sets in the eye-tracking task, while the remaining set served as lures in the recognition memory task. Eye-movements were recorded during the computer-based presentation of slides, showing three images at a time on a grey-scaled background (Figure 1). In each of the 40 trials, two images were presented at the upper half of the screen and one in the center of the lower half of the screen. Each trial contained one positive, one negative and one neutral image of the same age-category.

Arrangement and order of stimuli were pseudo-randomized. More specifically, it was assured that each type of valence would occur equally often in the three positions. Moreover, the three images presented together were selected so that differences in the average picture salience were minimized to control for differences in low-level image features [47]. Finally, trials were arranged in a way that not more than three succeeding slides belonged to the same age category. Each trial started with a central fixation cross for a jittered interval of 3–6 s. Subsequently, the three images were presented for 10 s (see Figure 1). Participants were instructed to look naturally at the pictures as if they were watching them on TV. After 20 of the 40 trials, a short break was provided to prevent drops in attention. A practice phase of four trials preceded the experiment. The whole procedure took approximately 20 minutes.

Eye movement data were continuously recorded with a sampling rate of 1000 Hz using an infrared pupil-corneal reflection technique (EyeLink 1000, SR Research Ltd., Ottawa, Canada). The head location was fixed using a chin rest and a forehead bar. The software Presentation (Neurobehavioral Systems) was used to present the picture stimuli on a 20-inch LCD monitor (Samsung SyncMaster 204B; display dimension = 40.64 cm×30.48 cm; resolution = 1600×1200 pixels; refresh rate = 60 Hz). Participants viewed the screen from a distance of 47 cm.

For the analysis, eye movement data were parsed into saccades and fixations using EyeLink's standard parser configuration, which classifies an eye movement as a saccade when it exceeds 30°/sec velocity or 8000°/sec^2^ acceleration. Subsequently, horizontal and vertical coordinates of fixations were drift corrected with reference to the central fixation cross at trial start. The baseline consisted of the average gaze position during the last 300 ms before stimulus onset. If the baseline was unavailable (e.g. due to blinks) or invalid (horizontal or vertical deviation of more than 100 pixels or 3 *SDs* from the average of all baselines in one session), it was replaced by the average baseline of all valid trials within the session. Such adjustment was necessary in 193 of all 2000 trials across participants. Finally, two measures were derived from the eye movement recordings: the number of fixations as well as the cumulative fixation duration on positive, negative and neutral pictures. The numbers of fixations were divided by the total number of fixations and the durations were divided by the total fixation time (excluding blinks and saccades) in each trial.

#### Singleton task

After the eye-tracking task, participants performed a visual search task. This singleton distraction paradigm was adopted from Costello and colleagues [48] and implemented using Cogent 2000 stimulus presentation software (Wellcome Department of Imaging Neuroscience, London, UK) in MATLAB (Mathworks Inc). Most importantly, this procedure allows for the assessment of participants' general ability to recruit top-down control trial-by-trial in order to focus on or to ignore salient visual stimuli (“singleton-detection mode”). In detail, participants had to indicate as quickly as possible whether a target-shape (circle) contained either the symbol “+” or “−” by pressing the left or right arrow key on a standard computer keyboard with the index and ring finger of the dominant hand. Key assignment was counterbalanced across participants. The target shape was surrounded by 3 or 11 distractors (squares). Please note that we focused our analyses on the 12-items-trials since we were mainly interested in control ability under high cognitive demands [48]. The 4-items-trials were left in the design to prevent participants from fatigue.

In the baseline blocks, 50% of the trials contained no particularly salient item (all green, i.e. no singleton), while in the other 50% of trials, one distractor was printed in red (singleton distractor). Baseline blocks started with the information that the target-circle is always green. Thus, participants could completely concentrate on one color, thereby minimizing the effect of the singleton distractor. In contrast, in the interesting singleton-detection blocks, participants were informed that in some cases (∼8%) the color singleton could also be the target. In other words, in addition to trials with no singleton and singleton distractors, there were also some trials in which the target-shape was printed in red (singleton target). Now, the color singleton could not be easily ignored but became highly salient because it might include the target relevant feature.

The whole task comprised eight blocks of 48 trials each. Baseline blocks (n = 4) were alternated with singleton-detection blocks (n = 4). Trials within the blocks were randomized with respect to display size (4 versus 12 items) and with targets and singletons appearing equally often in each quadrant of the visual field, while distances between singleton and target were balanced. Before and after half of the trials in each block, a break of 5000 ms was provided. Within this break, a note appeared on the screen for 3000 ms instructing/reminding the participant which type of block would follow (“Which symbol is displayed in the circle? The circle is always green” or “Which symbol is displayed in the circle? The circle can sometimes be red”).

The diameter of the circle was 1.2° and the sides of the squares were each 1.2°. All shapes were presented as color outlines against a black background on a 20-inch LCD monitor (Samsung SyncMaster 204B) with a viewing distance of approximately 60 cm. The shapes were presented in an invisible 2×2 grid (8.5°×8.5°) for balancing spatial locations of display items. In each trial, half of the shapes framed a plus and the other half an equal symbol. Shapes were separated by at least 0.61°. If participants did not respond within three seconds or responded incorrectly, a note appeared on the screen for 1000 ms, telling them to either “press quicker” or denoting “error”. The note was followed by a random delay of 200–800 ms. Following a correct response, a black screen was displayed for a random time interval between 1200–1600 ms before the next trial was initiated.

32 practice trials preceded the experiment. Completion of the task took approximately 20 minutes. One older participant was excluded from this particular analysis due to technical problems.

**Figure 2:**
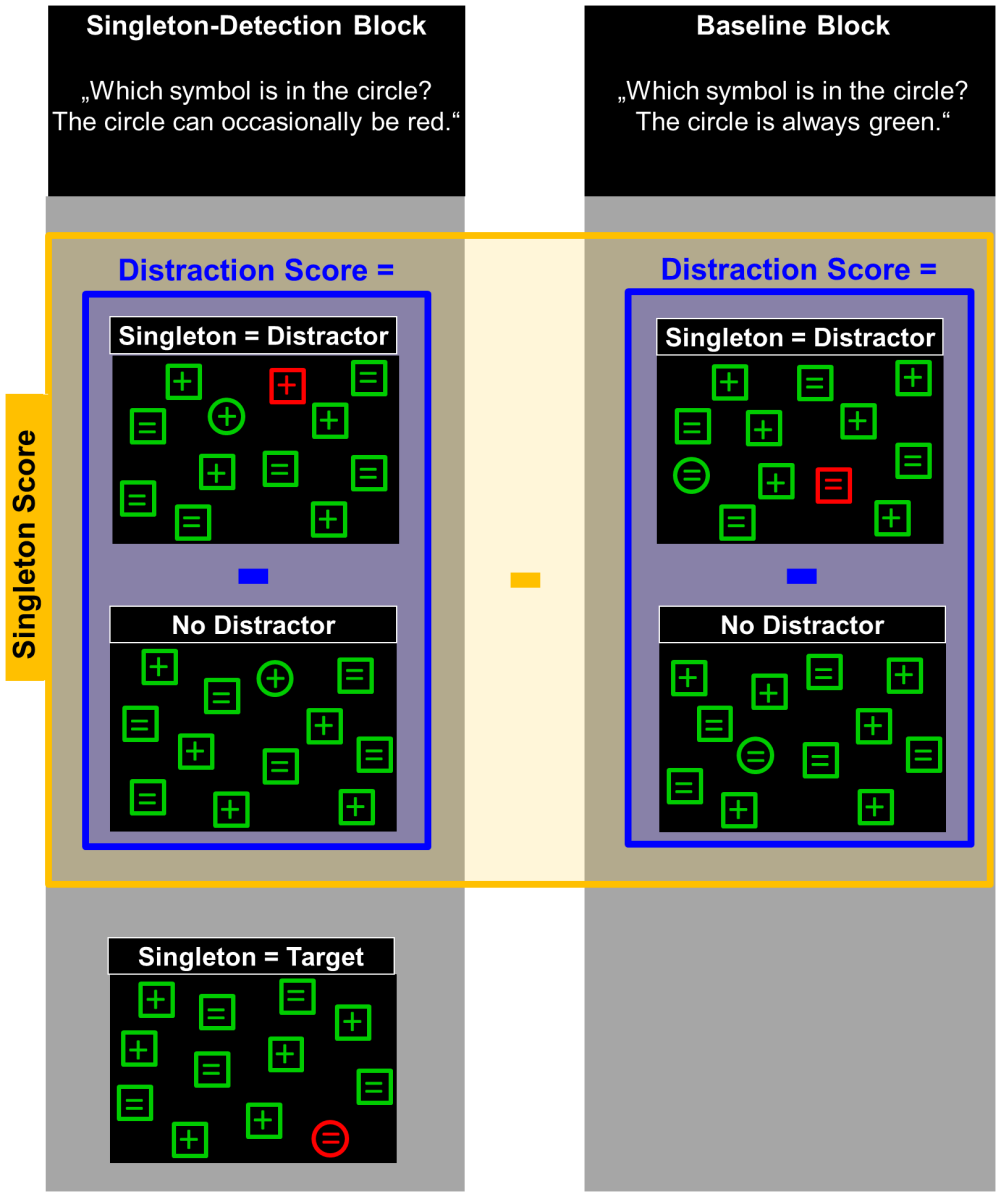
Illustration of the possible singleton-task conditions and of the score calculation. The left panel exemplifies the singleton-detection condition, in which the singleton distractor (red shape) can occasionally correspond to the target (circle). In the baseline condition, demonstrated in the right panel, the distractor never appears as the target. For both conditions, a distraction score (illustrated in blue) was calculated as the difference in reaction times between trials with singleton distractor and trials without distractor. Subtraction of these scores formed the singleton score (illustrated in yellow).

For the analysis of the singleton task, all trials in which participants missed a trial, responded inaccurately or faster than 200 ms were excluded. Trials in which the singleton corresponded to the target were also excluded due to specific response patterns induced by such trials (see [48] for details). In accordance with Costello et al. [48], we then calculated a distraction by singleton score for each participant for each condition (singleton detection vs. baseline). More specifically, mean reaction time in trials without singleton was subtracted from mean reaction time in trials with singleton distractor. In the next step, we estimated the ability to flexibly exert top-down control in the context of salient stimuli (which could be targets or distractors) by calculating the difference between singleton-detection and baseline distraction scores (adapted from Costello et al. [48]). Figure 2 provides an overview of the different task conditions and illustrates the calculation of the singleton score.

#### Recognition Memory and Affective Rating Task

In the recognition paradigm applied on the second day, the 120 stimuli presented on the first day were randomly intermixed with the 120 new stimuli from the second stimulus set. In a self-paced manner, participants had to respond to each image individually and indicate whether they had seen it on the previous day by pressing the corresponding button for “sure yes”, “probably yes”, “probably no”, or “sure no”. Such detailed response categories (instead of simple old/new ratings) were offered to reduce response bias and to maintain deep elaboration throughout the recognition task. Participants had five practice trials before the actual recognition test started and the whole test lasted approximately 40 minutes.

For the analysis of the recognition data, we collapsed across probable and sure responses because of the low and variable number of trials in each category. Subsequently, we calculated corrected hit rates by subtracting the proportion of false alarms from the proportion of hits. These values were computed for each stimulus category (valence by age-content) separately.

Finally, participants rated the affective quality of all stimuli with a computerized version of the Self-Assessment Manikin (SAM; [49]). The SAM is a non-verbal self-report measure, consisting of two bipolar five-point scales, which represent the affective dimensions of valence (ranging from unpleasant to pleasant) and arousal (ranging from calm to excited). Picture sequence was randomized for each participant and again five practice trials were provided, amounting to an overall duration of 45 minutes.

The recognition as well as the affective rating task were implemented using the software Presentation (Neurobehavioral Systems). Stimuli were presented on a 20-inch LCD monitor (Samsung SyncMaster 204B) with a viewing distance of approximately 60 cm.

### Data processing

All statistical analyses were accomplished with R, an open-source language for statistical computing (www.r-project.org). Fixation and recognition memory data were analyzed in 2×3×2 repeated measures analyses of variance (ANOVA), including the factors age-group (young/old), emotion (positive/negative/neutral) and image category (own-age/other-age). Where appropriate, degrees of freedom were adjusted using the Greenhouse-Geisser procedure [50] to correct for potential violations of the sphericity assumption. Two sample *t-tests* were applied in post-hoc comparisons between groups and paired *t-tests* for comparisons within groups. Pearson product moment correlations were used to calculate correlations between fixation data and the singleton score. Cohen's *d* and partial eta squared *η_p_^2^* are depicted as effect sizes for pair wise comparisons and ANOVAs, respectively.

## Results

### Ratings

As expected, ANOVAs for the SAM ratings including the factors emotion (positive/negative/neutral) and age-category of the image (young-age/old-age) as within-subject factors and group (young/old) as a between-subjects factor revealed significant main effects of emotion for the valence, *F*(2,96) = 203.54, ε = .61, *p*<.001, *η_p_^2^* = .81 as well as for the arousal scale, *F*(2,96) = 118.65, ε = .95, *p*<.001, *η_p_^2^* = .71. Post-hoc analysis indicated that these effects were driven by the following rankings for the valence scale: positive > neutral > negative and for the arousal scale: negative > positive > neutral (all *p*<.01). Results of interactions between emotion and age-category of the image revealed no significant group differences for none of the two SAM-scales (all *p*>.29).

### Eye-tracking

**Figure 3:**
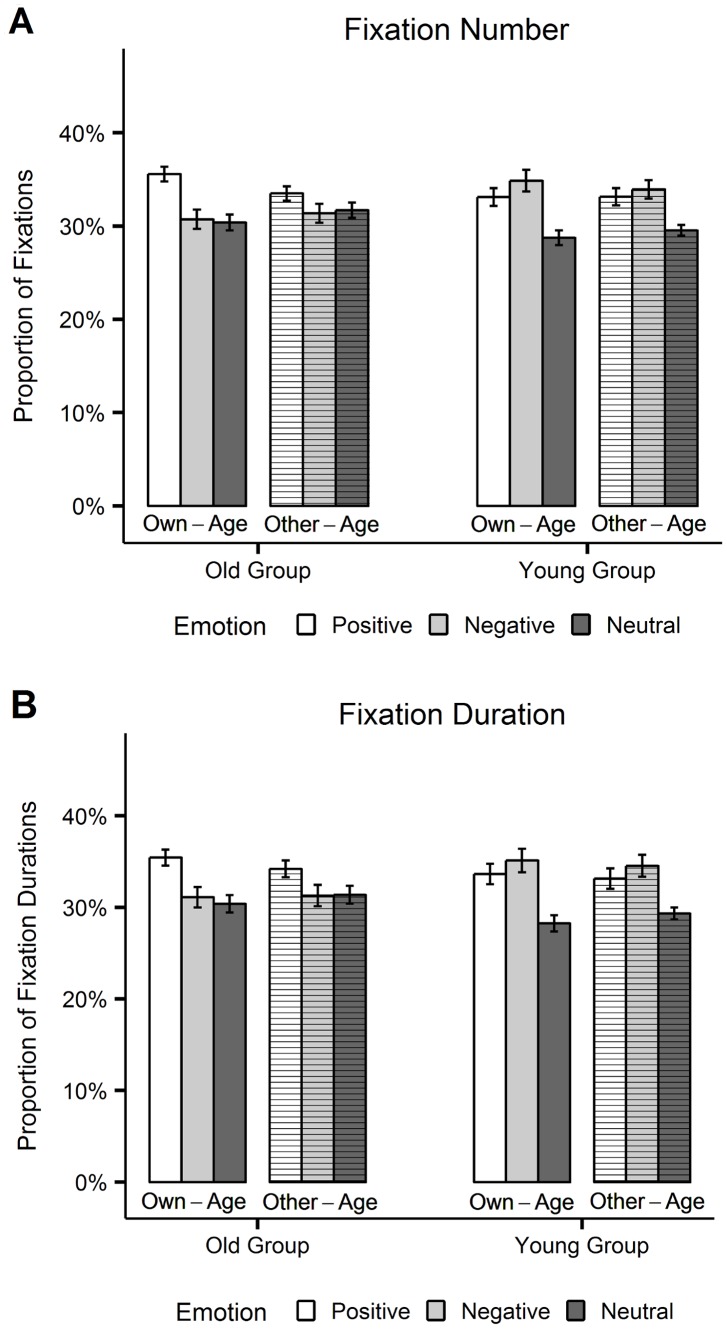
Eyetracking data. Proportions of fixations (A) and fixation durations (B) on positive, negative and neutral own-age and other-age stimuli, plotted separately for each age-group. Error bars represent standard errors of the mean.

For the analysis of the eye-tracking data, the relative number of fixations (Figure 3A) and the relative fixation durations (Figure 3B) were analyzed in separate repeated measures ANOVAs with emotion (positive/negative/neutral) and image category (own-age/other-age) as within-subject factors and group (young/old) as a between-subjects factor. For both models, a significant main effect of emotion was found (fixation number: *F*(2,96) = 7.19, ε = .87, *p*<.01, *η_p_^2^* = .13; fixation duration: *F*(2, 96) = 7.11, ε = .87, *p*<.01, *η_p_^2^* = .13), as well as a significant emotion x group interaction (fixation number: *F*(2,96) = 4.11, ε = .87, *p*<.05, *η_p_^2^* = .08; fixation duration: *F*(2,96) = 3.53, ε = .87, *p*<.05, *η_p_^2^* = .07). Moreover, for the number of fixations, a trend was observed for the emotion x image category interaction, *F*(2,96) = 2.96, ε = .97, *p* = .06, *η_p_^2^* = .06, as well as the three-way interaction of all factors, *F*(2,96) = 2.49, ε = .97, *p* = .09, *η_p_^2^* = .05.

**Figure 4:**
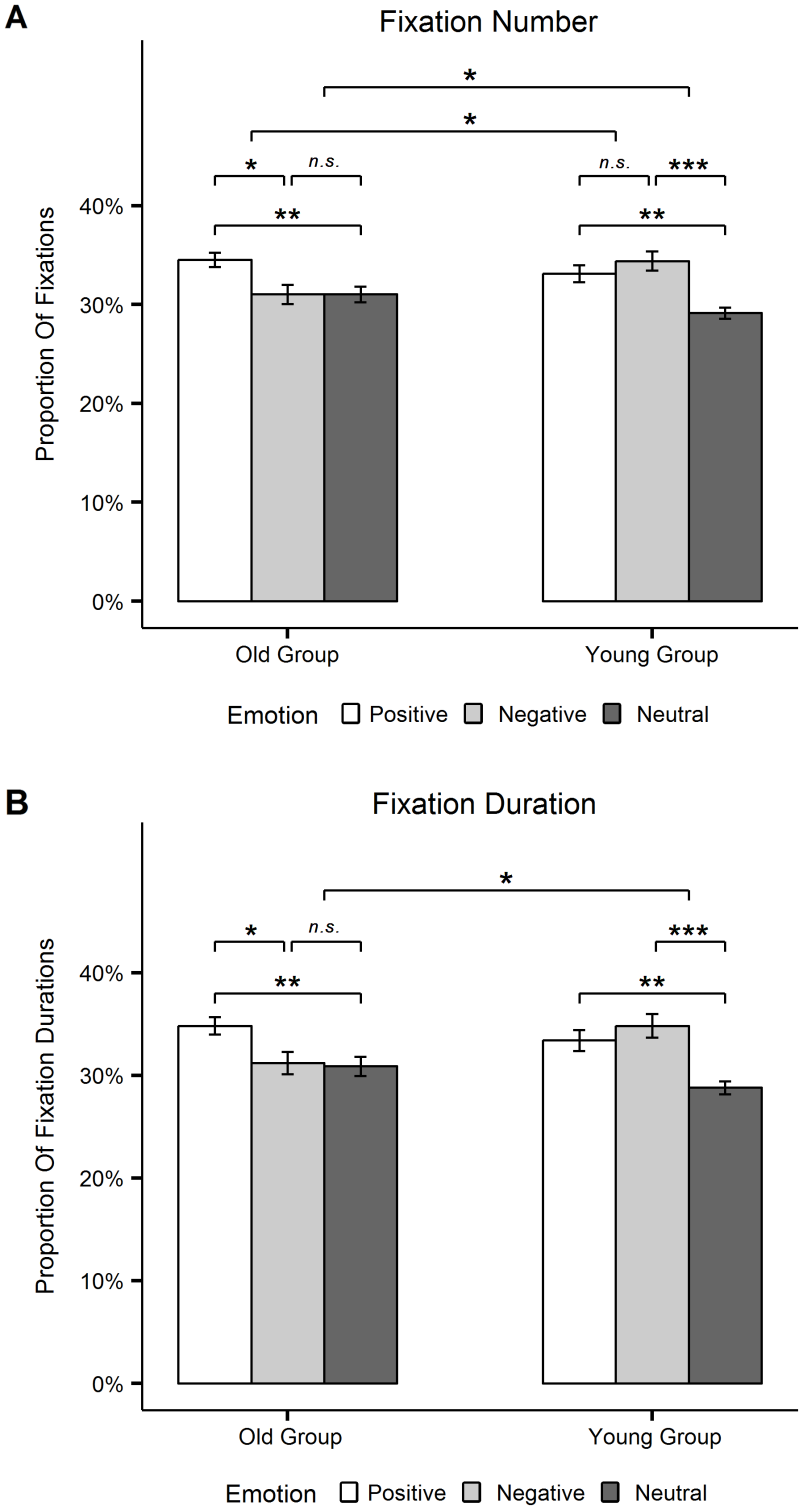
Post-hoc eyetracking results of emotion and emotion x group effects. Proportions of fixations (A) and fixation durations (B) on positive, negative and neutral stimuli aggregated across own-age and other-age stimuli, plotted separately for each age-group; Error bars represent standard errors of the mean. * *p*<.05, ** *p*<.01, *** *p*<.01, *n.s.* = not significant.

Post-hoc comparisons for the number of fixations across both age-categories (Figure 4a) indicated that older adults fixated positive stimuli significantly more than negative, *t*(24) = 2.31, *p*<.05, *d* = 0.46, and neutral ones, *t*(24) = 2.97, *p*<.01, *d* = 0.59, while in contrast, younger adults showed significant preferences for positive over neutral, *t*(24) = 3.68, *p*<.01, *d* = 0.74, and negative over neutral stimuli, *t*(24) = 3.91, *p*<.001, *d* = 0.78. The same pattern was observed for fixation durations across both age-categories (see Figure 4B): Older adults fixated positive stimuli significantly longer than negative, *t*(24) = 2.09, *p*<.05, *d* = 0.42, and neutral, *t*(24) = 2.80, *p*<.01, *d* = 0.56, stimuli, while younger adults significantly preferred positive, *t*(24) = 3.58, *p*<.01, *d* = 0.72, and negative, *t*(24) = 3.99, *p*<.001, *d* = 0.80, over neutral images. Moreover, direct group comparisons of these preferences revealed that for both, fixation numbers and fixation durations, the difference in negative versus neutral stimuli was significantly lower in older than in younger participants: *t*(48) = 2.50, *p*<.05, *d* = 0.71 (fixation numbers), *t*(48) = 2.40, *p*<.05; *d* = 0.68 (fixation durations). Additionally, the older adults' preference in their number of fixations for positive over negative stimuli was significantly greater than in younger participants, *t*(48) = 2.07, *p*<.05, *d* = 0.58.

**Figure 5:**
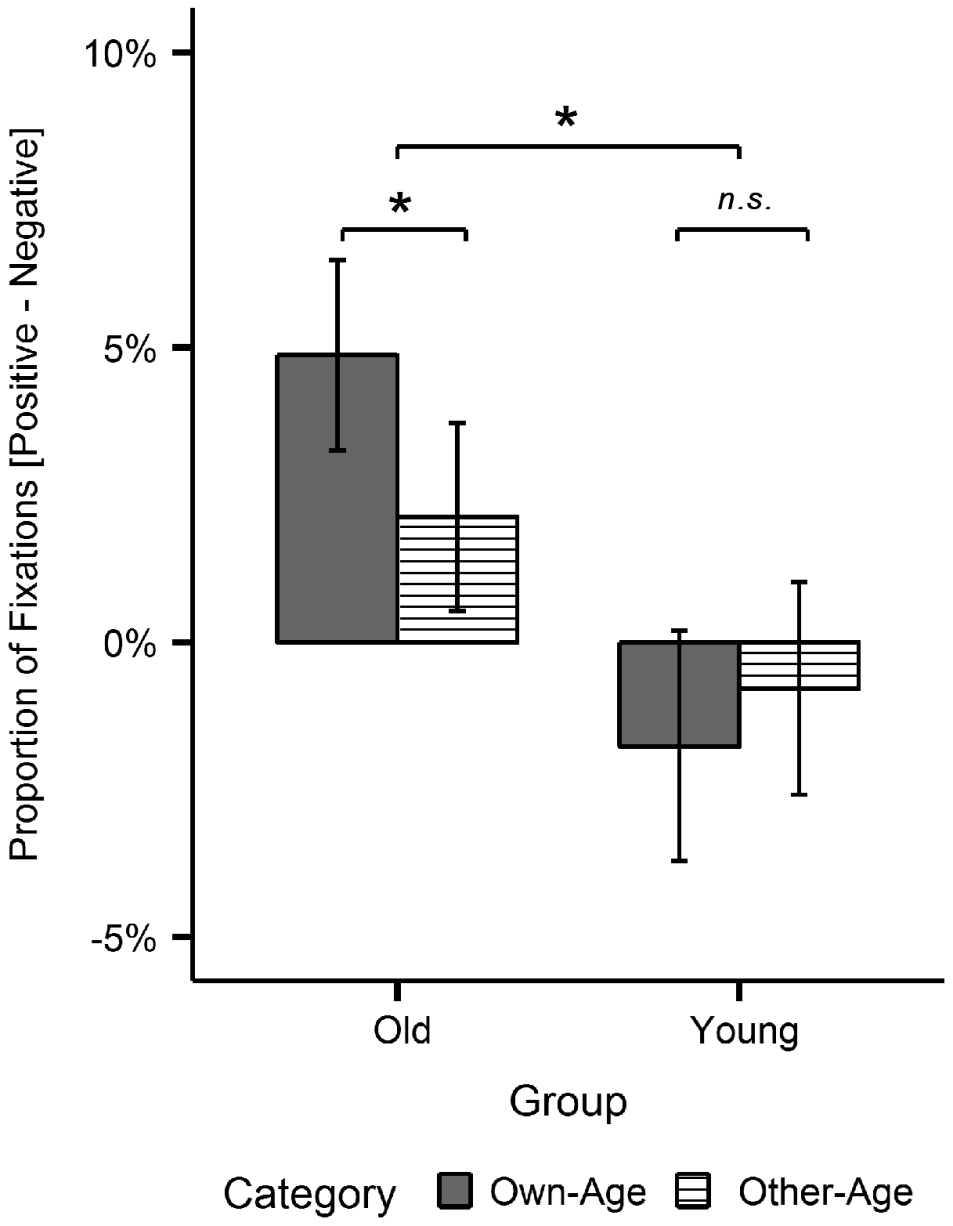
Post-hoc test results for interactions with image category. Difference between proportions of fixations for positive minus negative images in the two image categories (own-age vs. other-age), plotted separately for each age-group. Error bars represent standard errors of the mean. * *p*<.05, *n.s.* = not significant.

Regarding the marginally significant 3-way interaction for fixation number, post-hoc comparisons revealed that this trend was mainly driven by group x image category effects for positive versus negative stimuli. More specifically, older participants' focus on positive versus negative stimuli was significantly stronger for stimuli of the own-age compared with the other age-category, *t*(24) = 2.60, *p*<.05, *d* = 0.52, and this effect was significantly stronger than in younger participants, *t*(48) = 2.10, *p*<.05, *d* = 0.59 (Figure 5).

### The ability of top-down control and associations with the positivity effect in attention

Only few trials had to be discarded from the singleton analysis due to errors or missing responses (old group: *M* = 1.14%, *SD* = 1.09%; young group: *M* = 2.39%, *SD* = 2.02%).

First of all, we compared the singleton score between young and old adults. A two-sample t-test revealed no significant difference, *t*(47) = 1.44, *p* = .16.

**Figure 6:**
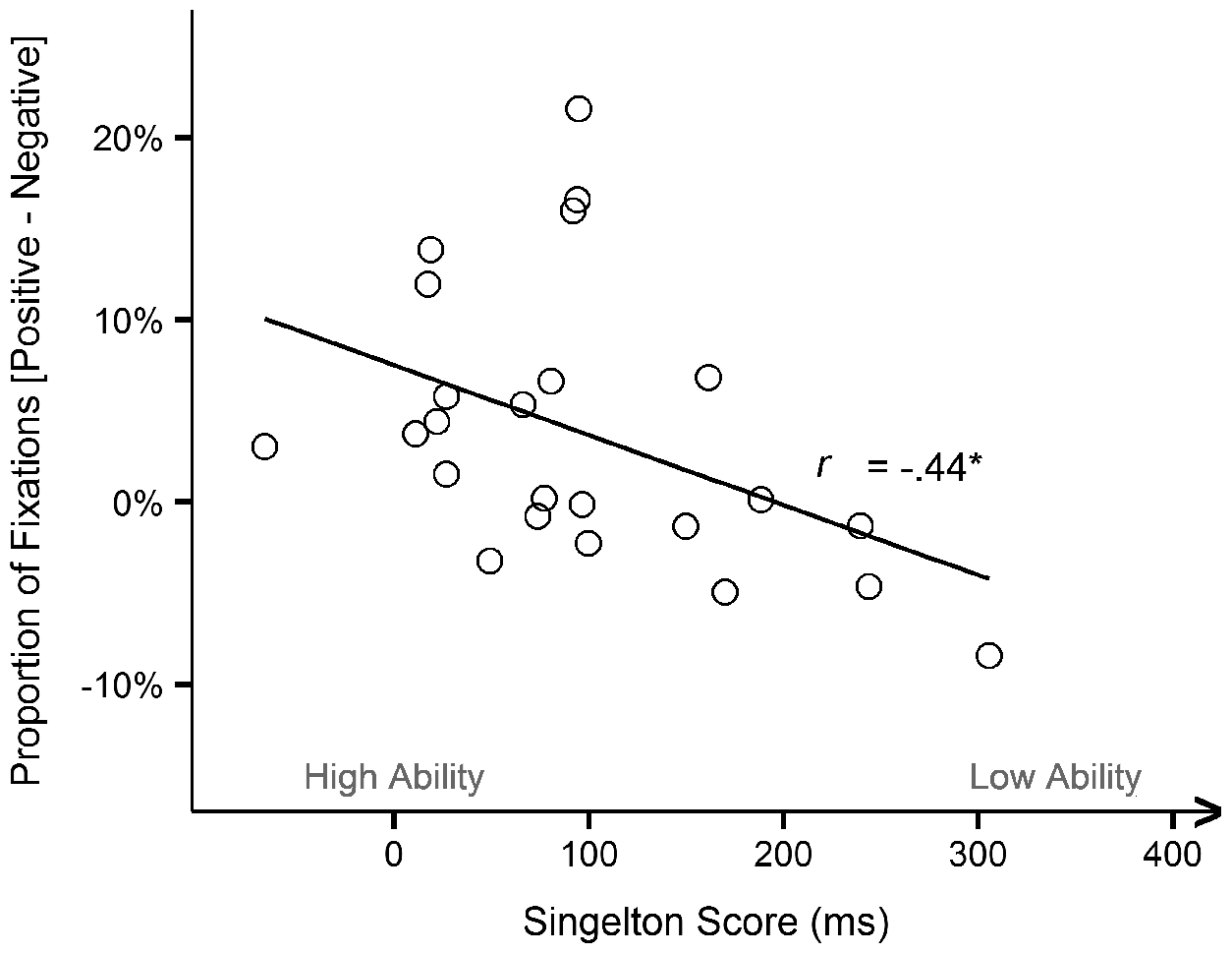
Correlation between the positivity effect and the singleton score. Illustrated for the number of fixations, but similar results were obtained for the fixation duration. * *p*<.05.

In the next step, we investigated whether there is a relationship between older participants' attentional PE (i.e. focusing on positive while ignoring negative stimuli) and their ability to flexibly exert top-down control. Pearson product moment correlations were calculated between older adults' singleton score and the number and duration of fixations on positive minus negative stimuli. Results revealed significant correlations with both eye-tracking measurements: *r* = −.44, *n* = 24, *p*<.05 for number of fixations and *r* = −.46, *n* = 24, *p*<.05 for fixation duration. As visualized in Figure 6, older participants who were more able to adaptively control their attention in the context of salient stimuli, showed a larger positivity effect.

### Recognition memory

Corrected hit rates were subjected to an ANOVA with the factors emotion (positive/negative/neutral), image category (own-age/other-age) and group (young/old). The analysis revealed significant main effects of emotion, *F*(2,96) = 8.12, ε = .97, *p*<.01, *η_p_^2^* = .15, and group, *F*(1,48) = 12.16, *p*<.01, *η_p_^2^* = .20, a significant interaction between emotion and group, *F*(2,96) = 4.07, ε = .97, *p*<.05, *η_p_^2^* = .08 as well as a significant interaction between emotion, image-category and group, *F*(2,96) = 3.29, ε = .98, *p*<.05, *η_p_^2^* = .06.

**Figure 7:**
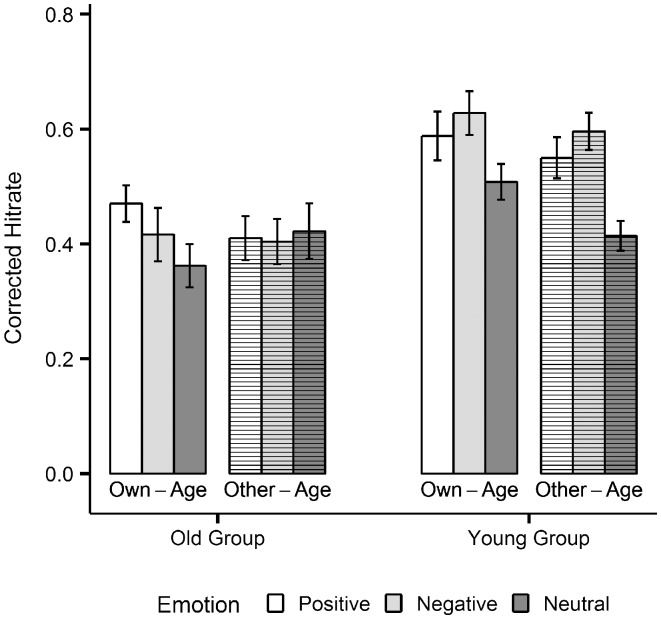
Memory data. Corrected hit rates for positive, negative and neutral own-age and other-age stimuli, plotted separately for each age-group. Error bars represent standard errors of the mean.

**Figure 8:**
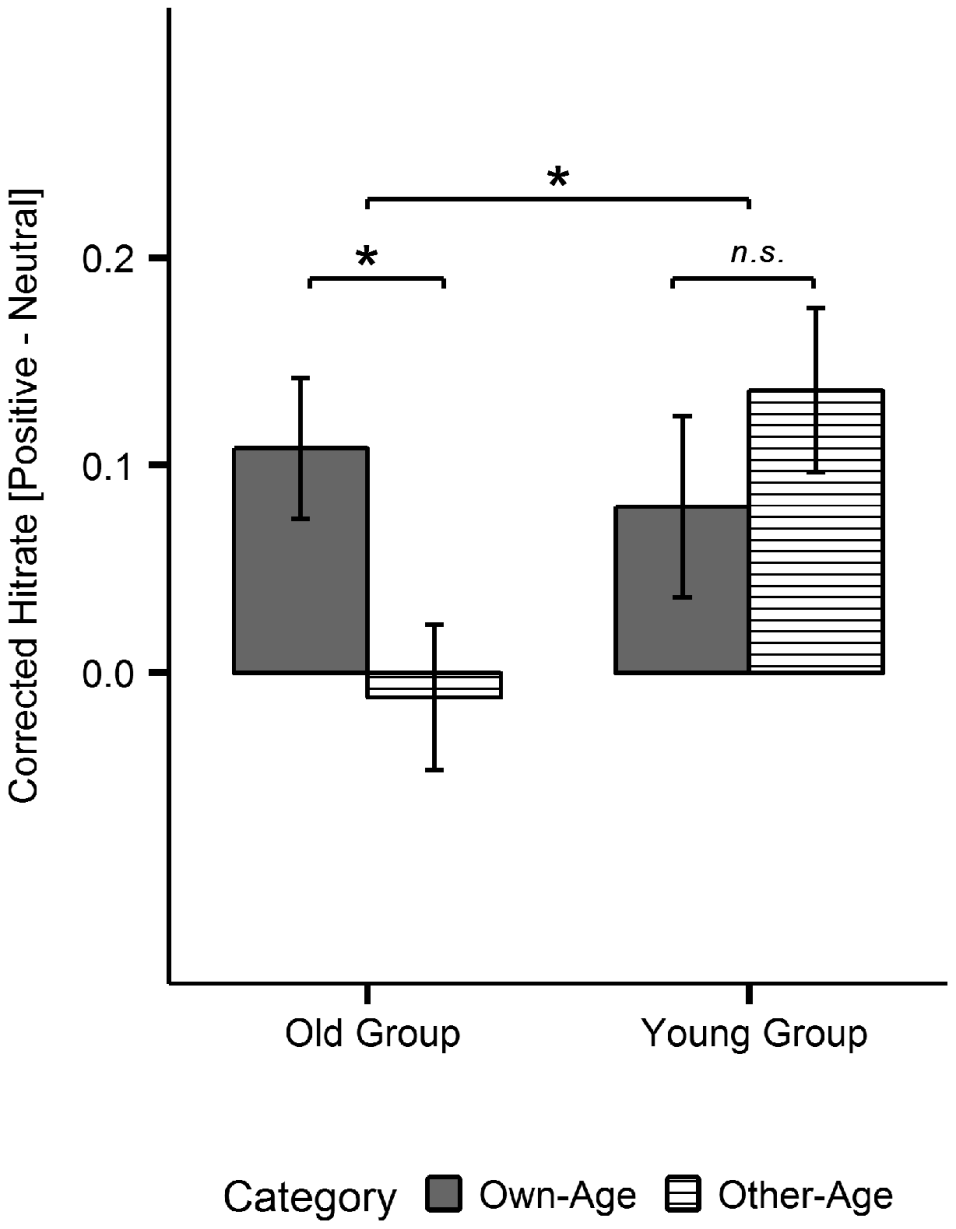
Post-hoc memory results for the three-way interaction between emotion, image category and age-group. Difference between corrected hit rates for positive minus neutral own-age and other-age stimuli, plotted separately for each age-group. Error bars represent standard errors of the mean. * *p*<.05, *n.s.* = not significant.

As evident in Figure 7, younger participants showed a general emotional memory enhancement for positive, *t*(24) = 3.40, *p*<.01, *d* = 0.69, and negative, *t*(24) = 4.23, *p*<.001, *d* = 0.83, over neutral items across both age-relevance conditions. In contrast, the emotional memory enhancement in the old group was restricted to positive over neutral own-age stimuli, *t*(24) = 3.19, *p*<.01, *d* = 0.65. Moreover, post-hoc analyses revealed that the latter effect was also the main reason for the significant 3-way-interaction between emotion, image category and group: the enhancement of positive over neutral memory for own-age versus other-age stimuli was significantly stronger in old compared to young adults, t(48) = 2.62, *p*<.05, *d* = 0.74 (see Figure 8).

## Discussion

Our study could demonstrate that older adults engage in selective attention to produce an age-specific positivity effect (PE) in attention. The selective depth of stimulus elaboration was also reflected in an analogical positivity preference in memory on the next day. Moreover, focusing on positive and ignoring negative stimuli was strongest in older adults with a generally higher ability of attentional control in the context of highly salient information. Finally, the observed PE was boosted for stimuli with higher own-age relevance, which further underlines older adults' selectivity to allocate cognitive resources in the service of prioritized emotional goals.

The main aim of the present approach was to further illuminate the most frequently discussed conditions and mechanisms underlying the PE in older adults' emotional attention and memory. In this context, one of the main hypotheses we tested was that the PE results from an age-related increased emphasis on selective attention as a tool to control emotional information processing. To study this question, we established an eye-tracking paradigm in which participants were simultaneously presented with positive, negative and neutral stimuli. Thus, participants needed to engage in selective attention in order to focus on a preferred emotion and to ignore non-target emotions respectively. Replicating and strengthening previous results (for a recent meta-analysis on the reliability of the effect see [1]), we found that older adults exhibited a PE, i.e. significantly focused their attention on positive and away from negative stimuli compared with young adults. In detail, while young adults showed a general boost in attention by emotional stimuli, older adults showed such a boost only for positive pictures, but seemed to ignore the saliency of negative stimuli. Intriguingly, a similar pattern was observed in memory for the presented stimuli on the next day, thus emphasizing the impact of selective processing on the depth of encoding. Life-span theorists have proposed that the occurrence of a PE is triggered by an increased resource allocation in the service of emotional goals to maintain emotional well-being in aging. In line with that, an increased focus on the positive has only been found in emotionally healthy older adults, but not in adults with late-life depression [24], and has been directly related to emotional well-being [6]. It has been argued that the prioritization of selective attention among different emotion regulation strategies is promoted by the age-related availability of underlying brain resources. In particular, there is evidence that while dorsolateral frontal brain regions are typically affected by an age-related decline, ventromedial circuits, including the anterior cingulate cortex (ACC), remain relatively preserved in late-life [51]. Thus, it could be speculated that elderly people focus on those strategies that are easiest to implement. Consistent with this idea, healthy older adults are often worse than younger adults [52] when instructed to use higher-order, rather dorsolaterally mediated regulation strategies, such as reappraisal [53], [54]. In contrast, they often demonstrate unimpaired or even better performance than younger adults when they get the opportunity to use selective inhibition and enhancement [55], which are thought to rely more on ventromedial brain regions [26]. In line with that, in our previous work we found an age-specific increase of ventromedial brain activity when healthy older adults selectively focused on positive stimuli [6] or inhibited negative information [24].

In contrast to most existing eye-tracking studies [8], [9], [33], [56], participants in our study were free to shift their attention towards preferred and away from unfavored emotional stimuli. Previous findings on instructed emotion regulation in older adults indicate that the PE in aging most likely occurs when attentional deployment is not controlled or instructed, and when there is an alternative stimulus on which older adults can focus their attention while ignoring negative information [19]. The lack of one or both of these aspects may account for the failure to observe age-specific emotional preferences in some previous studies [57] and their presence in our study might have maximized the effect.

Despite the aforementioned speculations on the role of preserved brain circuits in aging, it is still surprising that older adults are able to voluntarily shift their attention in the context of highly salient distraction as in our paradigm. There is ample evidence that cognitive functions including executive control are typically affected by an age-related decline [36]. However, most of these findings are limited to non-emotional distractors, while ignoring negative distractors is frequently maintained and associated with lower cognitive costs in older as compared to younger adults [5], [22], [23], [58], [59]. One discussed reason for this phenomenon is that older adults may be less sensitive to negative stimuli, for instance due to changes in amygdala functioning [38], and are consequently less challenged by ignoring salient (negative) information. This argument, however, cannot explain findings regarding a selectively increased distractibility by positive stimuli [6], [7] and the reported lack of age-effects in initial emotional experiences [10], [56]. A probably more convincing explanation is provided by the Socio-emotional Selectivity Theory, which proposes that the maintenance of well-being becomes a major goal in late-life and that cognitive resources are selectively recruited in the service of this goal. More specifically, when older people are confronted with emotional stimuli and have enough freely available cognitive resources, they predominantly activate emotion regulation in a goal-consistent manner, even when it is not required by the setting [6]. Following this assumption, older people with more cognitive resources should be particularly capable to produce a PE. In line with this hypothesis, we investigated whether there is a direct relationship between elderly peoples' selective attention ability and their tendency to exhibit a corresponding PE. Results revealed a significant correlation between older adults' attentional focus on positive compared to negative stimuli and their general ability to ignore highly salient distractors in a cognitively demanding setup. The ability to exert top-down control was measured with an established visual search task [48]. This task is able to assess the specific function of spontaneously focusing on target information in the context of salient distraction, which is probably involved when people voluntarily control their attention in an emotional context. The observed correlation in our study strongly supports this assumption and extends previous findings on links between cognitive ability and a PE in memory [12], [60].

Interestingly, emotionally healthy older adults in our study did not differ from young adults in their ability to manage distraction by highly salient stimuli. Although this finding is consistent with some previous findings [48], [61]–[63], it is not yet clear whether it reflects preservation, compensation, or even decline in older adults. For instance, it could again be speculated that a reduction in the responsiveness to arousing stimuli because of age-related limbic brain decline might facilitate the disengagement from salient but irrelevant stimuli [38]. While this might hold for our observed singleton findings, it cannot explain older adults' increased focus on highly salient, positive stimuli. In addition, and as discussed in the next section, the amplification of the PE through stimulus relevance rather supports assumptions from the “cognitive control hypothesis” [35] than from the “aging brain model” [38].

We further explored the idea that healthy older adults actively apply cognitive resources for emotional means by manipulating the personal relevance of the stimulus material. In fact, there is evidence that the selective allocation of cognitive resources in aging can be facilitated by self-referential processes and that older people predominantly engage in self-referential processing to compensate for age-related cognitive decline [25], [64]–[66]. Along these lines, we expected an amplification of the PE when older participants are presented with stimulus material that depicts social scenes with a higher degree of own-age relevance. Consistent with this expectation, we could show that elderly participants' emotional selectivity in attention and memory was enhanced for images of higher own-age relevance. Thus, our results fit with previous neuroimaging studies, demonstrating more engagement of the “emotion-regulation” brain network in older adults when the emotional material was processed under conditions of stronger in-depth elaboration [39], such as self-referent processing [29] and semantic elaboration [66].

Some previous studies that manipulated self-relevance by introducing own- and other age face stimuli in young and old adults did not demonstrate an enhanced PE for relevant stimuli [7], [40], [67]. One main reason for these results may be the comparatively lower degree of socioemotional relevance conveyed by decontextualized emotional faces as compared to more natural social scenes [68], [69]. Social emotional stimuli have a strong impact on self-referential processing as people try to understand the mental state of the other [70]. This is particularly pronounced the more similar the person is to the self [43], [44]. Other findings that are controversial to our results come from Tomaszczyk and colleagues [71]. More consistent with us, they used complex IAPS images but presented them in a single-stimulus encoding paradigm. The authors observed a reduction of the PE in memory under conditions of high compared to low personal relevance, suggesting that, here, the saliency signalled by the self-relevant negative stimuli interfered with emotional goals in the elderly. As mentioned above, the PE in aging most likely occurs when attentional deployment is not controlled and when there is an alternative stimulus on which older adults can focus their attention while ignoring negative information [19]. Both factors were lacking in the previous study, but present in our one, which suggests that they constitute significant modulators of the PE in aging.

The selection and categorization of our stimulus material was based on independent raters and validated by a sample of young adults. Thus, we cannot exclude the possibility that older adults' relevance ratings would differ from those of younger adults. In the same vein, to investigate the impact of personal relevance on participants' information processing in more detail, it seems desirable to consider individual relevance ratings. The fact that we still observed significant effects of the age-relevance, however, supports the assumption that we rather under- than overestimated the effect by this limitation. Furthermore, we only included emotionally healthy young and old adults in our study and can thus only speculate about the clinical role of our findings. Nevertheless, it has consistently been demonstrated that elderly patients with emotional disorders, such as geriatric depression or geriatric anxiety disorder, show marked executive control dysfunctions together with fronto-limbic abnormalities [24], [72]–[74]. Moreover, these patients are typically more strongly distracted by negative stimuli, which is paralleled by decreased prefrontal attention control activity [75], [76]. Taking these findings into account, we propose that the ability to selectively recruit cognitive control processes to assist emotional well-being might be an important resilience factor for emotional health in aging. Intervention- and prevention schemes might therefore benefit from including specific trainings of cognitive control and such training might be facilitated through the implementation of self-relevant material.

To summarize, our findings first of all strengthen prior research about the involvement of cognitive control in the PE. In addition, we could demonstrate that such emotional preferences in aging can be boosted through greater self-relevance of the stimuli. Overall, our findings highlight the importance to carefully control for both factors cognitive impact/resources and self-relevance in future studies on age-differences in emotional information processing.
